# Biochemical and Functional Characterization of the Trace Amine-Associated Receptor 1 (TAAR1) Agonist RO5263397

**DOI:** 10.3389/fphar.2018.00645

**Published:** 2018-06-21

**Authors:** Stefano Espinoza, Damiana Leo, Tatyana D. Sotnikova, Mohammed Shahid, Tiina M. Kääriäinen, Raul R. Gainetdinov

**Affiliations:** ^1^Fondazione Istituto Italiano di Tecnologia, Department of Neuroscience and Brain Technologies, Genoa, Italy; ^2^Department of Neurosciences, University of Mons, Mons, Belgium; ^3^Institute of Translational Biomedicine, Saint Petersburg State University, Saint Petersburg, Russia; ^4^Orion Pharma, Nottingham, United Kingdom; ^5^Orion Pharma, Turku, Finland; ^6^Skolkovo Institute of Science and Technology, Moscow, Russia

**Keywords:** TAAR1, RO5263397, BRET, ERK, CREB, anti-depressant

## Abstract

Trace amine-associated receptor 1 (TAAR1) is a G protein-coupled receptor, which signals through elevating intracellular cAMP levels, and expressed in most vertebrates, including rodents and humans. In recent years, several lines of evidence indicated the role of TAAR1 in the regulation of dopaminergic system and its importance in physiological processes such as locomotion, control of emotional states and cognition. In our study, we used RO5263397, a selective TAAR1 agonist, as a tool and characterized its pharmacology *in vitro* in HEK293 cells and its effects *in vivo* in tests assessing potential antidepressant and antipsychotic actions. We found that RO5263397 not only increases cAMP levels at very low concentrations but also can induce the phosphorylation of ERK and CREB in a concentration- and time-dependent manner. Like other TAAR1 agonists, RO5263397 potently suppressed high dopamine-dependent hyperactivity in mice lacking the dopamine transporter. Moreover, RO5263397 produced a strong antidepressant-like effect in the forced swim test comparable to fluoxetine. Furthermore, the antidepressant-like activity was blocked by pretreatment with SCH23390 (dopamine D1 receptor antagonist) or NBQX (glutamate AMPA receptor antagonist) but only in part by WAY100635 (serotonin 5HT1A receptor antagonist). In conclusion, our study confirms some previous *in vitro* and *in vivo* findings in relation to the pharmacological effects of RO5263397 but more importantly provides new insight on intracellular signaling pathway and other neurotransmitter receptors modulated by TAAR1 receptor activation.

## Introduction

Trace amine-associated receptor 1 (TAAR1), along with the other members of the TAAR family, is a G protein-coupled receptor (GPCR) that was identified in 2001 by two independent groups (Borowsky et al., 2001; Bunzow et al., 2001). It is expressed in most vertebrates, ranging from zebrafish to rodents, non-human primates and humans (Berry et al., 2017). In the brain, it is localized in discrete regions containing the dopaminergic neurons of the ventral tegmental area (VTA), the serotonergic neurons of the dorsal raphe (DR), the amygdala, the prefrontal cortex and several subregions of the basal ganglia. In peripheral tissue, TAAR1 has been found in the pancreas (the β-cells), the stomach, the intestine and also in different type of leukocytes (Babusyte et al., 2013; Raab et al., 2016). By using transgenic TAAR1 knockout (TAAR1-KO) mouse lines or TAAR1 selective pharmacological tools (e.g., agonists: RO5263397, RO5166017; antagonist: EPPTB) several investigators have attempted to further the knowledge and understanding of TAAR1 physiology and its possible role in different pathology (Wolinsky et al., 2007; Revel et al., 2011; Black et al., 2016; Ferragud et al., 2017). Multiple lines of evidence suggest that TAAR1 plays a significant role in modulation of central dopaminergic neurotransmission and function, by influencing processes both at the levels of the dopaminergic cell bodies and the dopaminergic terminals in the dorsal and ventral striatum (Lindemann et al., 2008; Leo et al., 2014; Espinoza et al., 2015). The overall effect of TAAR1 activation is inhibitory as regards to the dopamine system. Indeed, TAAR1 may be able to directly modulate the function of specific receptor subtypes such as D2 dopamine receptors (Espinoza et al., 2015). With regards to dopaminergic system related potential therapeutic utility, TAAR1 agonists have demonstrated activity in animal models pertinent to schizophrenia and addiction (Revel et al., 2013; Pei et al., 2015). In addition to the involvement in dopaminergic function, TAAR1 is also expressed in the serotonergic neurons of the DR, and its activation attenuates the firing rate of these neurons (Revel et al., 2011). The possibility of impact on serotonergic neurotransmission has broadened with data showing that TAAR1 agonists exhibited antidepressant-like properties in rats and monkeys (Revel et al., 2013). While the effect of TAAR1 on dopamine system and on locomotor activity is well-established, the downstream neurochemical pathways or receptors involved in mediating the antidepressant-like effect of TAAR1 agonists remains to be investigated. At the level of cellular signaling, TAAR1 is a GPCR coupled to Gαs, thus its activation leads to an increase intracellular cAMP levels and downstream signal transduction. However, to what extent cAMP-independent signal transduction is engaged requires further clarification. For example, it has been shown that TAAR1 can also influence the β-arrestin2 signaling pathway, likely through an interaction with the D2 dopamine receptor (Espinoza et al., 2015; Harmeier et al., 2015).

In the present study, we aimed to further characterize the TAAR1-dependent behavioral effects in animals depicting a hyperdopaminergic state (dopamine transporter knockout, DAT-KO, mice) or exposed to forced swim stress using the most selective agonist RO5263397 with improved pharmacokinetic properties (Revel et al., 2013). In addition, we performed *in vitro* studies to assess TAAR1 mediated effect on TAAR1 signaling proteins downstream of cAMP and β-arrestin2 such as CREB and ERK.

## Materials and Methods

### Animals and Reagents

DAT-KO mice were generated as previously described (Giros et al., 1996). C57BL/6Jx129Sv/J hybrid WT and DAT-KO mice, 3–5 months old, of both sexes (with equal number of male and female animals per group) were used. Since no difference in effects were observed between males and females, the data from male and female mice were combined. Adult male Spraque-Dawley rats used in the forced swim test (FST) were purchased from Harlan (Netherlands).

All cell culture reagents and buffers were from Invitrogen (Carlsbad, CA, United States) and Sigma (St. Louis, MO, United States), and FBS from JRH Biosciences (Lenexa, KS, United States). Coelenterazine *h* was purchased from Promega (Madison, WI, United States). Plasmids containing the cDNA for the human trace amine receptor were obtained from the cDNA resource center at the University of Missouri-Rolla and the American Type Culture Collection (Manassas, VA, United States) and modified as described. Plasmid expressing mTAAR1 was a kind gift from Hoffman-La Roche (Basel, Switzerland). RO5263397 was synthetized at Orion Pharma (Finland) and confirmed for purity and structure verification.

### Cell Culture and Transfection of Cell Lines

Human embryonic kidney 293 cells (HEK293T) were maintained in Dulbecco’s modified Eagle’s medium supplemented with 10% (vol/vol) of FBS, 2 mM L-glutamine and 0.05 mg/ml of gentamicin at 37°C in a humidified atmosphere at 95% air and 5% CO_2_. Transient transfections were performed 24 h after cells seeding using lipofectamine 2000 protocol (Invitrogen). 5 μg of mTAAR1 or hTAAR1 and 4 μg of EPAC for each milliliter of transfection solution were used for the experiments (Barak et al., 2008). For the bioluminescence resonance energy transfer (BRET) experiments, 24 h after transfection, the cells were plated in poly-D-lysine coated 96-well microplates (well-assay plate with clear bottom, Fisher Scientific) at a density of 70,000 cells per well in phenol red free Minimum Essential Medium containing 2% of FBS, 10 mM Hepes, 2 mM L-glutamine. The cells were then cultured for an additional 24 h.

### Bioluminescence Resonance Energy Transfer (BRET) Measurement

Bioluminescence resonance energy transfer experiments were performed as described previously (Espinoza et al., 2013). RO5263397 and EPPTB powder was dissolved in DMSO at the concentration of 10 mM and then diluted in PBS to the desired concentration. For time course experiments, the plate was read immediately after the addition of RO5263397 and for approximately 20 min. In order to calculate the EC_50_ values, a concentration response curve was performed using different concentration of the agonist. To evaluate the antagonistic effect of EPPTB, the antagonist was added 5 min before RO5263397. All the experiments were conducted in presence of the phosphodiesterase inhibitor 3-Isobutyl-1-methylxanthine (Sigma) at the final concentration of 200 μM. Readings were collected using a Tecan Infinite instrument that allows the sequential integration of the signals detected in the 465 to 505 nm and 515 to 555 nm windows using filters with the appropriate band pass and by using iControl software. The acceptor/donor ratio was calculated as previously described (Espinoza et al., 2013). We used an EPAC BRET biosensor to monitor cAMP levels. With this sensor an increase in cAMP is reflected in a decrease in the BRET ratio. Curve was fitted using a non-linear regression and one site specific binding with GraphPad Prism 5. Data are representative of four independent experiments and are expressed as means ± SEM.

### Antibodies and Western Blot Analyses

The antiphospho-ERK1/2 (Thr-202/Tyr-204), anti-ERK, anti-phospho-CREB (Thr-34) and anti-CREB antibodies were purchased from Cell Signaling Technology (Beverly, MA, United States). To analyze effect of RO5263397 on TAAR1-mediated intracellular signaling events in HEK-293 cells, hTAAR1 or mTAAR1 was transiently expressed in the cells. After 24 of transfection, cells were treated with RO5263397 at concentration ranging from 0.01 to 100 nM (for concentration response experiment) or at the same concentration and then lysed at different time points (for time course experiments). Cells were lysed with RIPA buffer supplemented with protease (Roche Diagnostic) and phosphatase (Thermo Scientific) inhibitors. After 10 min of incubation on ice, lysates were centrifuged for 10 min at 13000 rpm and supernatants were collected for protein concentration assay (BCA protein assay kit, Thermo Scientific). 25 μg of protein extract were separated on 10% SDS/PAGE and transferred on nitrocellulose membrane. All primary antibodies were incubated overnight at 4°C. Appropriate peroxidase-conjugate secondary antibodies (Pierce) and chemiluminescent reagents (ECL detection reagent, Amersham) were used. For quantitative analysis, total proteins were used as loading controls for phosphoprotein signals. Results obtained with RO5263397 were normalized to respective vehicle controls.

### Locomotor Activity

Effect of RO5263397 on spontaneous locomotor activity of DAT-KO or WT mice was tested as described previously (Revel et al., 2011). Briefly, DAT-KO or WT mice were placed in the locomotor activity chambers (Omnitech Digiscan, Accuscan Instruments, Columbus, OH, United States) for 30 min and then were treated with either saline or RO5263397 at different doses and total distance traveled was measured by analyzing infrared beam interruptions for another 90 min.

### Forced Swim Test

Male Spraque-Dawley rats (230–270 g) were used in the FST. The test was performed in a glass cylinder (46 cm × 20 cm), with tap water 25 cm deep and +24–25°C. Water was refreshed after each test and it was performed over two consecutive days. On the first day, the rats were forced to swim for a 15-min period and immediately after that the first dose of test compound or vehicle was administered (5 ml/kg p.o.). The second drug or vehicle administration was on the next day, 23 h after the first dosing. One hour after the second administration and 24 h after the first swimming session, the rats were subjected to a second 5-min swimming session and immobility, climbing and swimming time were registered. Rats were considered to show immobility when animals were inactive and displaying minor movements with one limb only; climbing behavior was registered when the rats were actively climbing at the walls of the cylinder; finally, time spent swimming (horizontal activity) was measured as the remaining time after immobility and climbing have been subtracted from total test time. After each FST session, the rats were dried with towel and allow rest under heating lamp. RO5263397 (0.1, 1.0, and 10 mg/kg) formulations were prepared in 5% Tween 80 in sterile H_2_O. Each formulation was freshly prepared each day of dosing. In experiments examining the effects of dopamine D1 (D1) receptor antagonist SCH23390 (0.1 mg/kg, s.c.), glutamate AMPA receptor antagonist NBQX (10 mg/kg, s.c.), and serotonin 5-HT1A antagonist WAY100635 (1 mg/kg, s.c.) the antagonists were administered 10 min prior to RO5263397. The dose selection for SCH23390, WAY100635, and NBQX was based on previous work with these agents (Jordan et al., 2005; Snigdha et al., 2011). Statistical analysis was done by using one-way ANOVA followed by Dunnett’s *post hoc* test.

### Study Approval

All procedures involving animals and their care were carried out in accordance with the guidelines established by the European Community Council (Directive 2010/63/EU of September 22, 2010) and were approved by the Italian Ministry of Health (DL 116/92; DL 111/94-B).

### Data Analysis

The data are presented as means ± SEM and analyzed using a two-tailed Student’s *t*-test or one-way ANOVA followed by Dunnet’s multiple comparison test unless otherwise indicated. ^∗^*p* < 0.05; ^∗∗^*p* < 0.01; ^∗∗∗^*p* < 0.001.

## Results

### RO5263397 Increases cAMP Mediated BRET Signal in a Dose-Dependent Manner in HEK-293 Cells

**FIGURE 1:**
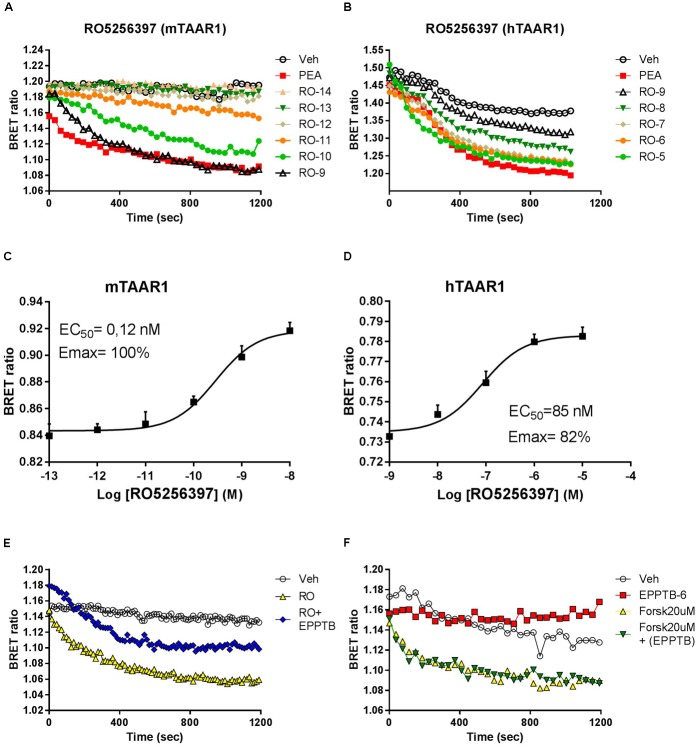
RO5263397 activates TAAR1 and increases cAMP-BRET signal. **(A,B)** HEK293 cells transfected with mTAAR1 (left panel) or hTAAR1 (right panel) were treated with increasing concentrations of RO5263397. cAMP is detected using a BRET biosensor where a decrease of a BRET ratio indicates an increase of cAMP levels. RO5263397 is added at the time 0 and then the plate was read for 20 min. We used as TAAR1 standard agonist β-phenyethylamine (PEA) at 10 μM. **(C,D)** Concentration response experiment. The data were represented using the log of the concentration and the curves were fitted using a non-linear regression in order to calculate the EC50. BRET signal was measured 15 min after the addition of RO5263397. **(E)** HEK293 cells transfected with mTAAR1 were pretreated with the TAAR1 antagonist EPPTB at 1 μM (or vehicle) and after 5 min with the agonist RO5263397 at 0.1 μM. To evaluate the specificity of EPPTB it was evaluated in combination with forskolin at 20 μM **(F)**. These panels are representative of three independent experiments.

To study the pharmacology of RO5263397, we first used an *in vitro* approach. Since TAAR1 is a GPCR coupled to stimulatory G protein, its activation evokes the production of cAMP. To monitor in real time TAAR1 activation, we transfected, in addition of mouse and human TAAR1, a cAMP BRET biosensor that changes the emission of the light according to the cAMP fluctuation (see section “Materials and Methods”). We then tested a range of concentrations of RO5263397 both in cells transfected with mouse or human TAAR1. As shown in **Figure 1**, RO5263397 increases cAMP-mediated BRET signal in a concentration dependent manner (**Figures 1A,B**), with no desensitization over the observation period. RO5263397 did not provoke a BRET signal in cells not transfected with TAAR1 (data not shown). Comparison with a maximal concentration (10 μM) of β-phenylethylamine (PEA), a known TAAR1 full agonist indicated, RO5263397 behaves as a full agonist at the mTAAR1 (EC_50_: 0.12 nM and Emax: 100%) and partial agonist at the hTAAR1 (EC_50_: 47 nM and Emax: 82%) as shown in **Figures 1C,D**, respectively. The data also indicate that RO5263397 shows species related difference with 392-fold higher potency at the mTAAR1 compared to hTAAR1.

### The TAAR1 Antagonist EPPTB Inhibits RO5263397 Effect

We further verified that the cAMP-mediated BRET signal seen with RO5263397 was due to activation of TAAR1 by pretreating the cells with the selective TAAR1 antagonist EPPTB. For these experiments, we used cells with mTAAR1 since EPPTB shows species selectivity mouse over human and rat. Pretreatment with EPPTB (1 μM) partially blocked the response to a maximal concentration (0.1 μM) of RO5263397 (**Figure 1E**). EPPTB alone did not affect BRET signal in mTAAR1 transfected cells. Furthermore, it did not affect the cAMP-BRET signal provoked by the adenylyl cyclase activator, forskolin (20μM) as shown in **Figure 1F**. These data indicate that mTAAR1 activation is necessary to observe the antagonist properties of EPPTB.

### RO5263397 Mediated ERK and CREB Phosphorylation in HEK293 Cells

**FIGURE 2:**
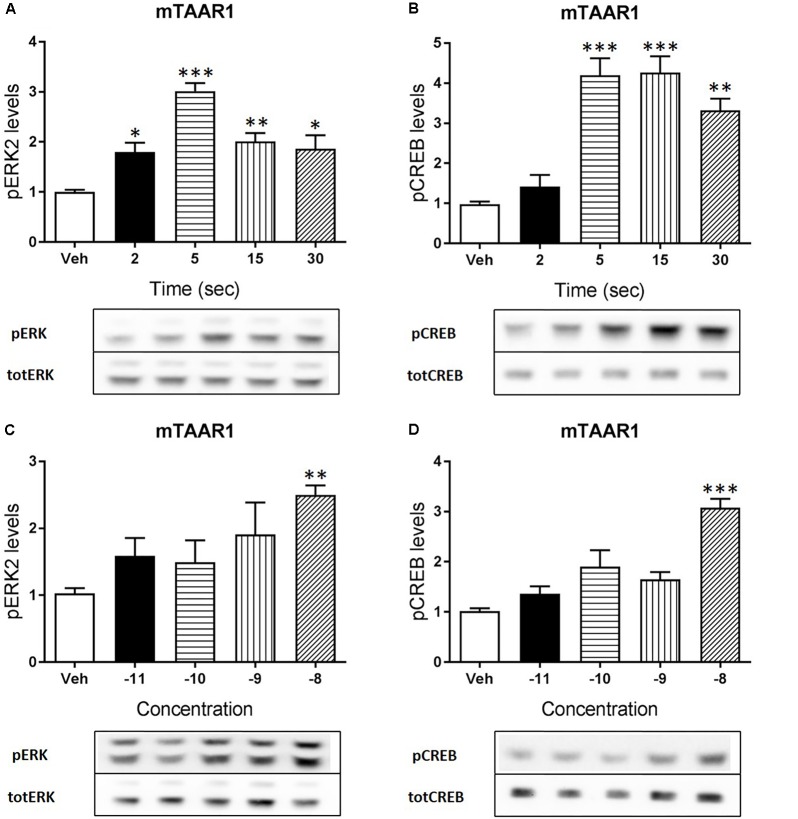
mTAAR1 activation leads to the phosphorylation of ERK and CREB. HEK293 cells transfected with mTAAR1 were treated with RO5263397 and then cells were washed and lysed at different time points to assess ERK **(A)** or CREB **(B)** phosphorylation. After the evaluation of the best time point at which RO5263397 induced an increase in pERK and pCREB, cells were treated with an increasing concentration of RO5263397 and lysed at 5 min for the evaluation of pERK **(C)** and at 15 min for the evaluation of pCREB **(D)**. Data represent means ± SEM. *N* = 3–5 per group. ^∗^*p* < 0.05; ^∗∗^*p* < 0.01; ^∗∗∗^*p* < 0.001. One-way ANOVA followed by Dunnet’s multiple comparison test.

**FIGURE 3:**
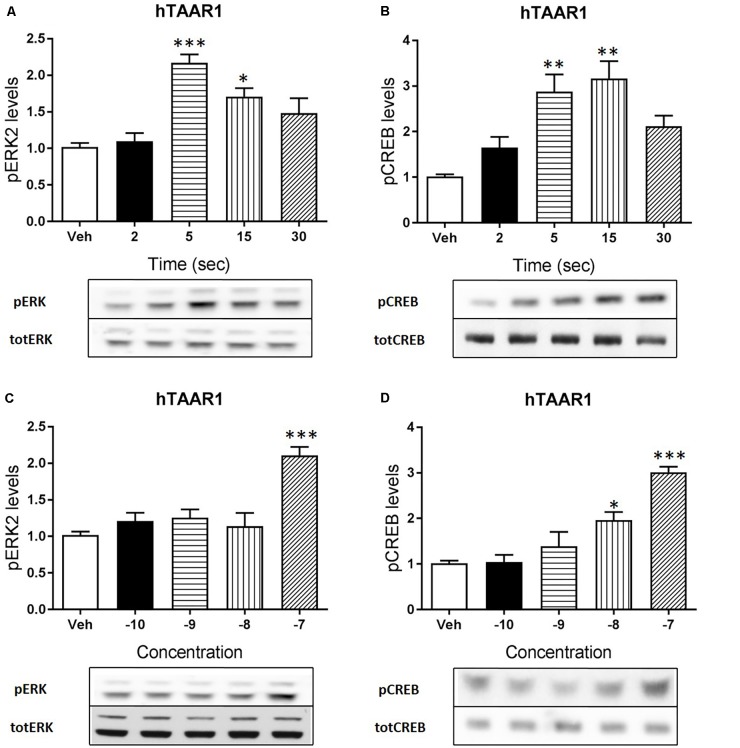
hTAAR1 activation leads to the phosphorylation of ERK and CREB. HEK293 cells transfected with hTAAR1 were treated with RO5263397 and then cells were washed and lysed at different time points to assess ERK **(A)** or CREB **(B)** phosphorylation. After the evaluation of the best time point at which RO5263397 induced an increase in pERK and pCREB, cells were treated with an increasing concentration of RO5263397 and lysed at 5 min for the evaluation of pERK **(C)** and at 15 min for the evaluation of pCREB **(D)**. Data represent means ± SEM. *N* = 3–4 per group. ^∗^*p* < 0.05; ^∗∗^*p* < 0.01; ^∗∗∗^*p* < 0.001. One-way ANOVA followed by Dunnet’s multiple comparison test.

Apart from cAMP induction, TAAR1 signaling could involve other proteins, such as β-arrestin2 and AKT/GSK3 pathways. However, cAMP and also β-arrestin2 are linked to downstream effectors such as CREB and ERK, and TAAR1 seems to modulate the activity of these two proteins. In order to verify this hypothesis, we used HEK293 cells expressing mTAAR1 or hTAAR1. First, we treated the cells expressing mTAAR1 with RO5263397 and we performed a time course at a concentration of the compound that was the first to reach the efficacy of 100%, according to our previous BRET experiments (10 nM for mTAAR1 and 100 nM for hTAAR1). As shown in **Figures 2A,B**, RO5263397 was able to induce the phosphorylation of ERK2 and CREB, with the maximum effect at 5 min for pERK and at 15 min for pCREB. After measuring the optimal time for the induction of pERK and pCREB, we performed a concentration response curve using different concentrations of RO5263397 (**Figures 2C,D**). We then repeated the same time course experiment in cells expressing hTAAR1, and obtained similar results (**Figure 3**).

### RO5263397 Inhibits Spontaneous Hyperactivity in DAT-KO Mice

**FIGURE 4:**
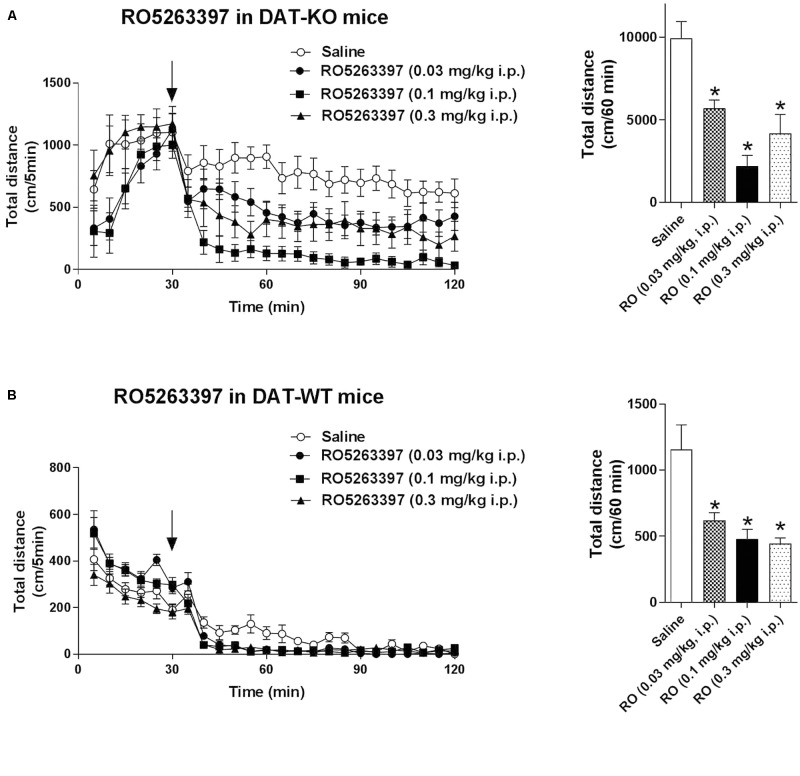
RO5263397 reduces the hyperactivity in DAT-KO mice. DAT-KO mice **(A)** or DAT-WT mice **(B)** were put in the locomotor activity cages to let them habituate for 30 min. Then, mice were treated with either saline or RO5263397 at different doses and total distance traveled was measured for another 90 min. The left panel represents the dynamics of effect over all 90 min of observation. The right panel indicates the cumulative total distance calculated over the first 60 min following drug administration. All the data are shown as mean ± SEM per group (*N* = 8–12 per group). ^∗^*p* < 0.05. One-way ANOVA [*F*(3,36) = 13.25, *p* < 0.001 for effect in DAT-KO mice **(A)**; *F*(3,28) = 9.645, *p* = 0.002 for effect in DAT-WT mice **(B)**] followed by Dunnet’s multiple comparison test.

Trace amine-associated receptor 1 agonists can reduce dopaminergic hyperactivation, for example after cocaine treatment (Revel et al., 2011). We tested the ability of RO5263397 to reduce the hyperactivity spontaneously present in DAT-KO mice. RO5263397 dose-dependently suppressed the locomotor activity in DAT-KO mice at all doses tested (0.03, 0.1, and 0.3 mg/kg, i.p.) with the most pronounced action noted at the dose 0.1 mg/kg (**Figure 4A**). In WT mice, RO5263397 also demonstrated significant locomotor-inhibiting action (**Figure 4B**).

### Antidepressant-Like Effect of RO5263397 Involves AMPA and D1 Receptors

**FIGURE 5:**
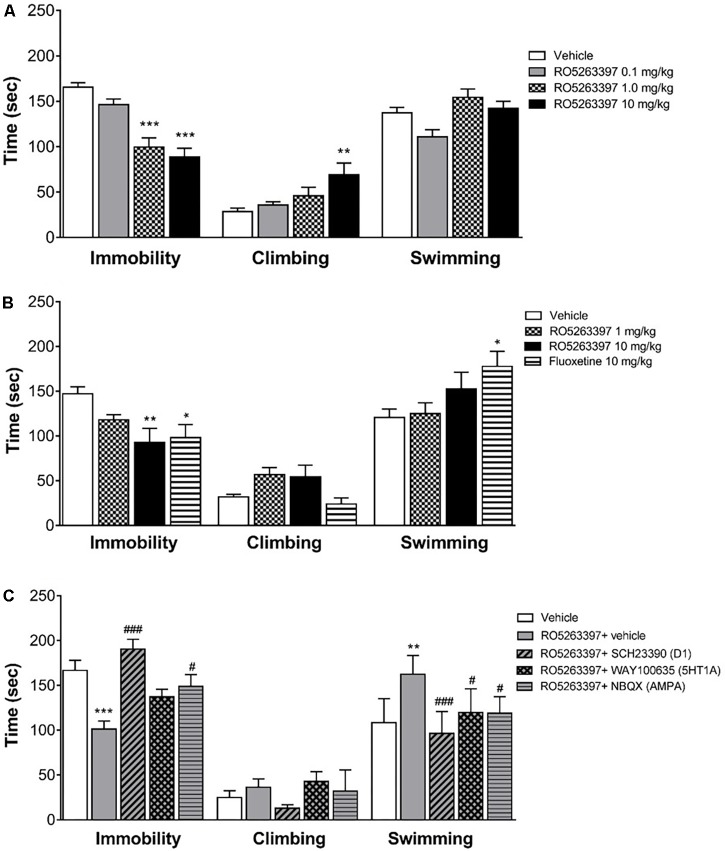
RO5263397 has antidepressant properties in the forced swimming test (FST) in rats. **(A)** Rats were treated p.o. with vehicle (5% Tween 80 in sterile H_2_O) or RO5263397 at different doses and then tested in the FST and time spent in immobility, climbing, and swimming behavior were recorded. Statistics (vehicle, RO5263397 three doses), Immobility: *F*(3,24) = 21.24, *p* < 0.0001, Climbing: *F*(3,24) = 4.523, *p* = 0.0119, Other: *F*(3,24) = 5.617, *p* = 0.0046. **(B)** The effect of RO5263397 and fluoxetine. Statistics (vehicle, RO5263397, and fluoxetine), Immobility: *F*(3,20) = 4.497, *p* = 0.0144, Climbing: *F*(3,20) = 3.713, *p* = 0.0285, Other: *F*(3,20) = 3.279, *p* = 0.0423. **(C)** The effect of RO5263397 was evaluated in the presence of SCH23390 (D1 receptor antagonist), NBQX (AMPA antagonist), and WAY100635 (5-HT1A antagonist). Statistics (vehicle, RO5263397, and antagonists), Immobility: *F*(4,30) = 10.82, *p* < 0.0001, Climbing: *F*(4,30) = 1.766, *p* = 0.1618, Other: *F*(4,30) = 8.481, *p* = 0.0001. All the data are shown as mean ± SEM per group (*N* = 6–8 per group). ^∗∗^*p* < 0.01, ^∗∗∗^*p* < 0.001 vs. Vehicle. ^#^*p* < 0.05, ^###^*p* < 0.001 vs. RO5263397.

Trace amine-associated receptor 1 selective agonists have multiple *in vivo* actions, affecting both the locomotor and emotional behaviors. However, while locomotor behavior is mostly mediated by the dopamine system, less is known about the neurobiological basis underpinning the TAAR1 effect on emotional behaviors. Thus, we tested RO5263397 in the FST to particularly examine the role of 5HT1A, AMPA or D1 receptors in its antidepressant-like activity in rats. RO5263397 significantly reduced immobility of rats at 1 and 10 mg/kg (p.o.), by 40 and 47%, respectively, compared to the vehicle group (**Figure 5A**). The lower dose of RO5263397 (0.1 mg/kg p.o.) had no significant effect on immobility time. RO5263397 significantly increased climbing behavior only at 10 mg/kg p.o., by 144%. At the lower doses 0.1 and 1 mg/kg RO5263397 increased climbing 26 and 62%, respectively although these effects were not significant. There was no effect on swimming activity at the doses examined. The RO5263397 provoked decrease in immobility time was similar in magnitude to fluoxetine (10 mg/kg, p.o.), although the latter drug also significantly increased swimming time (**Figure 5B**).

Then, to understand whether other neurotransmitters could be involved in the anti-depressant action of RO5263397, we treated rats with RO5263397 alone or in combination of antagonists of D1 receptor, 5-HT1A serotonin receptor and AMPA receptor. RO5263397 alone significantly reduced immobility time of rats at 1 mg/kg (p.o.), by 40% compared to the vehicle group (**Figure 5C**). When RO5263397 1 mg/kg was combined with D1 receptor antagonist SCH23390 (0.1 mg/kg, s.c.), immobility time was significantly increased, by 88% as compared to the effect of RO5263397 alone, 14% above the level of vehicle group. In combination with AMPA antagonist NBQX (10 mg/kg, s.c.), immobility time was also significantly increased, by 47% as compared to the effect of RO5263397 alone. 5-HT1A antagonist WAY100635 (1 mg/kg, s.c.) had no significant effect on RO5263397-induced decrease in immobility time. RO5263397 alone at the dose 1 mg/kg (p.o.) or in combination with D1, 5-HT1A or AMPA receptor antagonists did not have significant effect on climbing behavior. In this experiment, however, RO5263397 significantly increased swimming behavior which was attenuated by all three antagonists investigated.

## Discussion

In this study, we characterized the *in vitro* and *in vivo* pharmacology of a TAAR1 selective agonist RO5263397. TAAR1 is a GPCR coupled to Gαs and its stimulation by an agonist elicits an increase of cAMP inside the cells. This effect has been previously demonstrated in different heterologous systems, using TAAR1 from different species, including mouse, rat, human, and non-human primate (Borowsky et al., 2001; Bunzow et al., 2001; Miller et al., 2005; Barak et al., 2008). Since TAAR1 wild type form is expressed predominantly in the intracellular compartment, to better understand its pharmacology different groups adopted strategies to improve TAAR1 membrane expression, such as by adding small peptide to the N-terminal domain or by using chimeras with other GPCRs. In this study, we used a mTAAR1 with a leader sequence and a hTAAR1 version with the first nine amino acids of the β2-adrenergic receptor (Barak et al., 2008). We used a BRET based biosensor to monitor in real time the cAMP accumulation inside the cells induced by the TAAR1 agonist that have already proven to be extremely sensitive to cAMP variations (Salahpour et al., 2012). As expected, we observed that RO5263397 potently stimulated the cAMP production in HEK293 cells transfected with mouse or human TAAR1. EPPTB, a selective mouse TAAR1 antagonist, blocked the effects of RO5263397 confirming specificity of action. We found that RO5263397 behaves as a full agonist respect to mTAAR1 and as partial agonist respect to hTAAR1. This is partially in contrast with the original study that described this compound, since it has been reported that RO5263397 is a partial agonists for all TAAR1 species (Revel et al., 2013). The EC_50_ are comparable between our study and the study from Revel and colleagues, and also the maximal efficacy of RO5263397 in stimulating hTAAR1 (82% in our study vs. 81% of the original study). However, while in our hand RO5263397 is a full agonist in cells transfected with mTAAR1, Revel and collaborators showed that RO5263397 has a maximal efficacy of 59%. This discrepancy could be explained by the differences in assays used, Revel et al. (2013) measured cAMP accumulation with an immune assay kit while we monitor cAMP levels in real time in alive cells, and this could influence the evaluation of the receptor pharmacology. Indeed preliminary analysis with the same method as used by Revel et al. (2013) indicated that RO5263397 behaves as a partial agonist at human (Emax: 84%) and mouse (Emax: 31%) TAAR1.

Besides cAMP, TAAR1 signaling has been linked also to other pathways, such as β-arrestin2 dependent signaling cascade (Espinoza et al., 2015; Harmeier et al., 2015). Thus, we wanted to evaluate whether the activity of proteins linked to cAMP and β-arrestin2 such as CREB and ERK could be modulated by TAAR1 activation. We performed a time course and a concentration response experiments in HEK293 cells and observed that RO5263397 indeed induced the phosphorylation of CREB and ERK2. As already described, the induction of ERK is faster, with a peak at 5 min, while the activation of CREB is slower, accordingly to its nature of transcription factor. In a previous work, we showed that 3-metoxytyramine, a dopamine metabolite, is also a TAAR1 agonist and could induce the phosphorylation of ERK and CREB *in vitro* (Sotnikova et al., 2010). However, here we directly demonstrated with a selective agonist that TAAR1 activation is responsible for this effect. These data show how TAAR1 signaling can be different depending on the cellular environment and the potential co-expression with other receptors. We and others showed that in presence of D2 dopamine receptors TAAR1 cAMP signaling was reduced, and blockade of D2 receptor restores cAMP signaling (Espinoza et al., 2011). Moreover, when co-expressed with D2 dopamine receptors, TAAR1 affects also β-arrestin2 signaling, resulting in reduced D2 dopamine receptor- dependent GSK3β activation (Harmeier et al., 2015).

Evaluation in a variety of animal models indicates that TAAR1 agonists have a broad neuropsychopharmacologic profile with efficacy in a range of behavioral paradigms supporting potential therapeutic application in multiple disease areas (see Berry et al., 2017). They can reduce the dopaminergic activation induced by pharmacological treatments (e.g. phencyclidine, cocaine) or naturally present in transgenic animals (such as in DAT-KO mice) and enhance the effects of antidopaminergic drugs such as olanzapine (Revel et al., 2011). As it has been shown with other TAAR1 agonists, RO5263397 potently reduced dopamine-dependent hyperactivity in DAT-KO mice. These and other observations strongly support the potential of TAAR1 agonists as a novel treatment approach for a range of dopamine-related disorders such as schizophrenia, ADHD, and addiction.

At the same time, TAAR1 is expressed in the serotonergic neurons of the DR and can influence the activity of serotonergic system (Lindemann et al., 2008; Revel et al., 2011). RO5263397 was tested in rats and monkeys in different behavioral tasks and among other actions it has showed an antidepressant-like action (Revel et al., 2013). In the present study in rats RO5263397 dose-dependently reduced immobility time in the FST, confirming the results reported by Revel et al. (2013). In addition, we show that this may be, at least in part, due to facilitating active behavior such as climbing or swimming which have been suggested to be driven by increase in serotonergic and noradrenergic activity, respectively. Furthermore, using a pharmacological approach we investigated the involvement of specific dopamine, serotonin, and glutamate receptors in mediating the antidepressant-like effect of RO5263397. The rationale was based on evidence supporting the modulatory influence of TAAR1 on these neurotransmitter pathways and in particular the suggested relevance of the cortical dopamine D1, 5HT1A, and AMPA receptors in antidepressant action in the FST (Cryan et al., 2005; Browne and Lucki, 2013). Thus, dopamine D1, 5HT1A, and AMPA receptors have been proposed to be, at least partly, involved in mediating the antidepressant activity of noradrenaline reuptake inhibitors, serotonin reuptake inhibitors, and ketamine, respectively. We found that the D1 dopamine receptor antagonist SCH23390 and the glutamate AMPA receptor antagonist NBQX were effective in attenuating the reduction in immobility produced by RO5263397, whilst the serotonin 5-HT1A antagonist was less effective, reducing only the swimming behavior. The antagonists alone at the doses tested do not exhibit activity in the FST (Rogoz and Skuza, 2006; Koike and Chaki, 2014; Jastrzebska-Wiesek et al., 2016; Witkin et al., 2017). Previously, we showed that TAAR1 can directly interact with D2 receptors but not D1 receptors (Espinoza et al., 2011). Moreover, this TAAR1/D2 interaction could influence TAAR1-mediated effect on dopaminergic system, especially the striatal signaling and the locomotor behavior (Espinoza et al., 2015). Thus, it is possible that the effect of TAAR1 agonists on depressive behavior is mediated not by a direct interaction between TAAR1 and D1 receptors but likely by a functional effect on D1 receptors located in cortical areas that are responsible for anti-depressant action. These data provide new insight into the downstream neurotransmitter systems affected by TAAR1 receptor stimulation and suggest that it may be associated with an upregulation of tone at glutamate AMPA and dopamine D1 receptors.

Taken together, in this study we demonstrated that TAAR1 activation not only induce the increase of cAMP levels inside the cells, but also can induce the phosphorylation of two important signaling proteins, the MAP Kinase ERK and the transcription factor CREB. RO5263397 is highly effective *in vivo* in mice and rats demonstrating potential antipsychotic and antidepressant activity. Moreover, the antidepressant-like effect of the TAAR1 agonist RO5263397 relies on the engagement of other neurotransmitter systems, particularly involving the D1 receptor and the AMPA glutamate receptor.

## Author Contributions

SE performed experiments, designed research, analyzed data, and wrote the manuscript. DL performed experiments and wrote the manuscript. TS performed experiments and wrote the manuscript. MS, TK, and RG designed research, analyzed data, and wrote the manuscript.

## Conflict of Interest Statement

MS and TK were employed at Orion Pharma. The remaining authors declare that the research was conducted in the absence of any commercial or financial relationships that could be construed as a potential conflict of interest.
